# Development of Gelatin-Coated Microspheres for Novel Bioink Design

**DOI:** 10.3390/polym13193339

**Published:** 2021-09-29

**Authors:** Muskan Kanungo, Yale Wang, Noah Hutchinson, Emma Kroll, Anna DeBruine, Subha Kumpaty, Lixia Ren, Yuelin Wu, Xiaolin Hua, Wujie Zhang

**Affiliations:** 1 Biomolecular Engineering Program, Physics and Chemistry Department, Milwaukee School of Engineering, Milwaukee, WI 53202, USA; kanungom@msoe.edu (M.K.); krolle@msoe.edu (E.K.); debruinea@msoe.edu (A.D.); 2 Mechanical Engineering Department, University of Wisconsin-Milwaukee, Milwaukee, WI 53211, USA; yalewang@uwm.edu; 3 Biomedical Engineering Program, Electrical Engineering and Computer Science Department, Milwaukee School of Engineering, Milwaukee, WI 53202, USA; hutchinsonn@msoe.edu; 4 Mechanical Engineering Program Department, Milwaukee School of Engineering, Milwaukee, WI 53202, USA; kumpaty@msoe.edu; 5 School of Material Science and Engineering, Tianjin University, Tianjin 300072, China; lxren@tju.edu.cn; 6 Department of Obstetrics, Shanghai First Maternity and Infant Hospital, Tongji University School of Medicine, Shanghai 201204, China; wuyuelin_wyl@163.com

**Keywords:** pectin, electrospray, vascularization, gelatin, microspheres, hydrogel, bioink, scaffold

## Abstract

A major challenge in tissue engineering is the formation of vasculature in tissue and organs. Recent studies have shown that positively charged microspheres promote vascularization, while also supporting the controlled release of bioactive molecules. This study investigated the development of gelatin-coated pectin microspheres for incorporation into a novel bioink. Electrospray was used to produce the microspheres. The process was optimized using Design-Expert^®^ software. Microspheres underwent gelatin coating and EDC catalysis modifications. The results showed that the concentration of pectin solution impacted roundness and uniformity primarily, while flow rate affected size most significantly. The optimal gelatin concentration for microsphere coating was determined to be 0.75%, and gelatin coating led to a positively charged surface. When incorporated into bioink, the microspheres did not significantly alter viscosity, and they distributed evenly in bioink. These microspheres show great promise for incorporation into bioink for tissue engineering applications.

## 1. Introduction

According to the United States Health Resources and Services Administration, there are over 107,000 people on the national transplant waiting list, and 17 people die each day while waiting for a transplant [1]. While the need for organs has been increasing, the number of available organs is largely insufficient. Bioprinting is a tissue engineering approach that uses bioink containing cells and biomaterials to produce tissue and organs.

Bioinks that stimulate vascularization are of particular interest because vascular networks support cell viability and encourage structural organization, a significant feature for tissue engineering applications. Microspheres have been incorporated into bioinks to accomplish vascularization and release bioactive molecules in a controlled manner. Previous studies demonstrated that a scaffold with positively charged microspheres could promote vascularization when cultured with human umbilical vein endothelial cells (HUVECs) [2,3]. For instance, alginate-chitosan microspheres successfully led to vascularization within the collagen scaffold even without the incorporation of vascular endothelial growth factor (VEGF) [2]. An additional study explored the effect of positively charged chitosan-coated microspheres in the pectin-based bioink for both vascularization and estradiol sustained release [4]. However, the microsphere preparation process employed a double-emulsion system with a high degree of complexity, and the batch-to-batch variation in chitosan characteristics may cause inconsistencies in the final product [5]. Moreover, chitosan may pose a risk to mammals due to its immune-stimulating activities, as mammalians are unable to produce chitosan naturally [6]. Its poor mechanical strength necessitates crosslinking reactions, yet the resulting surface is unfavorable for cell attachment of certain cell types. The poor cellular affinity of chitosan is associated with a lack of cell-binding sites, limiting its application as a biomaterial. Thus, various extracellular matrix (ECM) molecules, like arginine-glycine-aspartic acid (RGD) tripeptides, have been immobilized on chitosan microspheres. These ECM molecules improve the material’s cellular affinity because their signaling domains specifically bind with integrins on cell membranes to enhanced cell attachment and proliferation [7].

Gelatin, a hydrolyzed form of collagen, is a natural biopolymer that displays potential in tissue engineering due to its exceptional biocompatibility and ability to promote cell adhesion and proliferation because of its RGD moieties [8]. Coating microspheres with gelatin could potentially promote cell adhesion and vascularization. A recent study showed that gelatin and gelatin-chitosan scaffolds are favored over chitosan-based scaffolds for bone tissue engineering applications in terms of biocompatibility [9]. In addition, the same study showed that gelatin can be modified or crosslinked to obtain the desired biochemical properties. The results indicated that both scaffolds made of gelatin and gelatin-chitosan crosslinked with glutaraldehyde had some effectiveness during bone regeneration [9]. Among the commonly used crosslinkers, 1-ethyl-3-(3-dimethylaminopropyl)carbodiimide hydrochloride (EDC) is a zero-length crosslinker that activates carboxyl groups to conjugate to amino groups, forming neutral amide (covalent) bonds and enhancing the mechanical stability of microspheres [10]. 

**Scheme 1:**
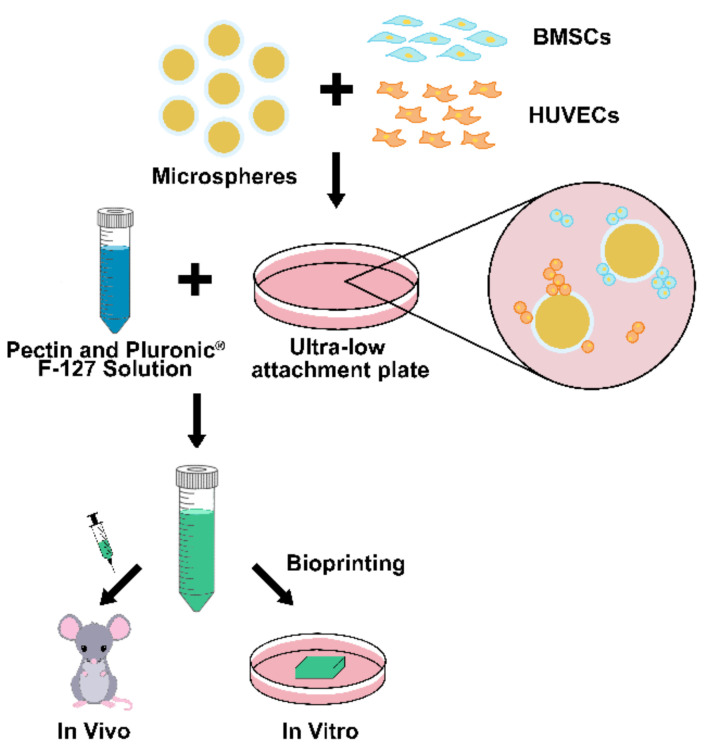
Applications of microsphere-incorporated bioink for fabrication of vascularized tissue.

This study aims to develop a novel bioink that incorporates gelatin-coated pectin microspheres with the potential to promote vascularization and controlled release of bioactive compounds. As shown in Scheme 1, gelatin-coated microspheres can be incubated with HUVECs (for vascularization) and functional cells, such as bone marrow mesenchymal stem cells (BMSCs). Microspheres/cells can be incorporated into the bioink for scaffolding. To keep the overall scaffold composition simple, pectin-based microspheres were chosen because pectin is also the major component of the base bioink developed previously. Pectin is primarily a linear polysaccharide found in the cell walls of plants, and it is comprised mainly of α-(1-4)-linked D-galacturonic acid residues with interspersed 1, 2-linked L-rhamnose residues [10]. Divalent ions (such as Ca^2+^ and Ba^2+^) cause crosslinking throughout the pectin molecules and allow hydrogel spheres to form from droplets through the formation of shifted “egg-box” structures when crosslinking low methoxyl (LM) pectin [11]. Pectin-based hydrogel systems have been used in drug delivery and tissue engineering applications, including the development of artificial red blood cells, due to their biocompatibility and biodegradability [12].

## 2. Materials and Methods

### 2.1. Materials

Low methoxy pectin was obtained from Willpowder (20.4% esterification degree, Miami Beach, FL, USA). Gelatin from porcine skin (G1890) and Pluronic^®^ F-127 (P2443) were purchased from Sigma-Aldrich (St. Louis, MO, USA). 1-ethyl-3-(3-dimethylaminopropyl)carbodiimide hydrochloride (EDC, 22980) and 2-morpholinoethanesulfonic acid (MES, M0606) were attained from Thermo Fisher Scientific. All materials were used as received.

### 2.2. Microsphere Preparation 

An electrospinning setup (Linari Engineering, Valpiana, Italy) was used to produce microspheres through electrospray. A freshly prepared pectin solution, 3.5–6% (*w*/*v*), was electro-sprayed into a 0.15 M CaCl_2_ solution for approximately 10 min. The microspheres were then collected by centrifugation (1200 rpm; 5 min).

### 2.3. Optimization of Microsphere Production Process 

Preliminary studies demonstrated that pectin solution concentration (A), voltage (B), flow rate (C), and distance between the needle tip and the surface of the gelation bath (D) were significant parameters and provided insight into what working ranges could be used for each factor (Table 1). Design-Expert^®^ software (Version 13; Stat-Ease Inc., Minneapolis, MN, USA) was used to optimize the microsphere production process. The Box-Behnken design (BBD) model was used. A total of 29 trials were performed based on the design. The responses for optimization were size, uniformity, and roundness. Size was measured using NIH ImageJ software. Uniformity and roundness were assessed on a scale of 1–10. The target size was <200 µm and the maximum uniformity and roundness rating was 10. A size of less than 200 µm was the aim for biocompatibility, mechanical properties, and bioprintability considerations [13].

Quadratic models were employed to represent the data, represented by Equation (1),

(1) $$Y=\beta_{0}+\overset{k}{\underset{i=1}{\sum }}\left(\beta_{i}X_{i}\right)+\overset{k}{\underset{i=1}{\sum }}\left(\beta_{i}X_{i}^{2}\right)+\overset{k-1}{\underset{i=1}{\sum }}\;\overset{k}{\underset{j>i}{\sum }}\left(\beta_{ij}X_{i}X_{j}\right),$$

where Y is the value of the response variable; $\beta_{0}$ is the intercept coefficient; the first $\beta_{i}$ items are linear coefficients; the second $\beta_{i}$ items are the quadratic coefficients; and $\beta_{ij}$ items are coefficients of interaction terms.

### 2.4. Modifications of Microsphere 

**Scheme 2:**
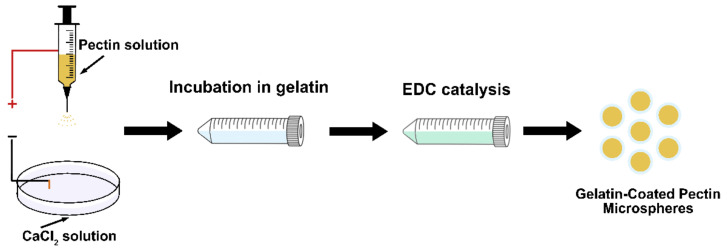
Process for producing gelatin-coated microspheres.

As shown in Scheme 2, the collected microspheres (calcium-pectin) were incubated in 0.5–2% (*w*/*v*) gelatin solutions for 15 min and rinsed twice in DI water. The microspheres were incubated overnight in EDC in MES buffer (15 mg/mL, pH = 4.8) at 4 °C. Microspheres were rinsed with DI water and placed in phosphate-buffered saline (PBS) for analysis under an inverted microscope (EVOS XL; Thermo Fisher Scientific, Waltham, MA, USA).

### 2.5. Characterization of Microspheres

Microspheres at each step of the production process—calcium-pectin microspheres (PM), microspheres after gelatin coating (GCM), and GCM after EDC catalysis (GCEM) —were characterized. The zeta potentials of different types of microspheres (suspended in DI water) were measured using a Zetasizer (Nano ZS; Malvern Instruments, Westborough, MA, USA). For scanning electron microscopy (SEM) imaging, microspheres were mounted onto an aluminum stub and sputter-coated with a 2 nm layer of iridium. Samples were examined under a Hitachi S-4800 ultrahigh-resolution cold cathode field emission scanning electron microscope (FE-SEM) at an accelerating voltage of 9.0 kV. Microspheres (oven-dried at 37 °C) were analyzed using Attenuated Total Reflection Fourier Transform Infrared (ATR-FTIR; MIRacle 10, IR-Tracer 100; Shimadzu, Kyoto, Japan) spectroscopy.

### 2.6. Characterization of Bioink 

A previously developed procedure was used to prepare a base bioink composed of 3% (*w*/*v*) pectin and 20% (*w*/*v*) Pluronic^®^ F-127 [14,15]. To prepare the microsphere-incorporated bioink, the microspheres were gently dispersed in the base bioink with a volume ratio of 1:50 (microspheres: base bioink). The kinematic viscosity of the bioink with and without microspheres was measured using a suspended level viscometer (Cannon Instrument Company; State College, PA, USA). The density was also determined by measuring the mass of 5 mL of bioink (density = mass/volume).

### 2.7. Scaffold Bioprinting Process

Allevi software (Philadelphia, PA, USA) was used to open the STL file for the object to be bioprinted. The bioink with microspheres (~25 °C) was loaded into the BioBot1 bioprinter (Allevi) and extruded through a 24-gauge blunt needle tip using a pressure of 10 psi and an axis movement velocity of 6 mm s^−1^. Bioink was extruded onto a Petri dish, with an AmScope Microscope Temperature Control Stage Slide Warmer (TCS-100; AmScope; Irvine, CA, USA) maintaining a temperature of 37 °C. Pluronic^®^ F-127 (present in the base bioink) gels when its temperature is greater than 30 °C, contributing to the gelation of the first few bioprinted layers [16]. After multiple layers were printed, the addition of warm (~37 °C) CaCl_2_ around the bottom of the scaffold cross-linked the pectin to form the permanent hydrogel structure.

## 3. Results and Discussion

### 3.1. Microsphere Production Process Optimization

**Figure 1:**
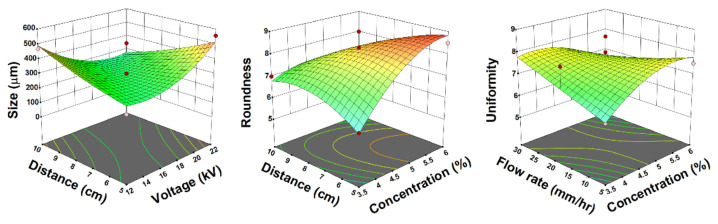
Surface response curves showing effects of most significant interaction on dependent variables (responses).

Three responses (size, roundness, and uniformity) were used for the optimization of the microsphere production process (Figure 1). The most important parameter impacting size was flow rate (*p* = 0.0039), with the general trend being that as flow rate increased, microsphere diameter increased, which is consistent with previous studies [17,18,19,20]. This is observed because with a larger the flow rate, more liquid is extruded through the syringe needle, yielding a larger droplet. The most significant interaction impacting size was that between voltage and distance (*p* = 0.0004). The relationship between voltage and size is supported by the concept of critical voltage. A sufficiently high voltage is required to overcome the surface tension of the droplet at the needle tip and to form small microspheres. The collection distance influences electric field strength. As the distance increases, the electric field decreases, resulting in larger microspheres [12,21,22,23]. Thus, the voltage has to be adjusted carefully with respect to distance.

Roundness is most significantly impacted by concentration (*p* = 0.0025). In general, microsphere roundness improves as polymer concentration increases over the working range due to a higher degree of chain entanglement [20]. The interaction between distance and concentration (*p* = 0.0045) also affects roundness strongly. To obtain spherical morphology, an adequate amount of time is needed for the droplet leaving the needle tip to obtain a spherical shape before contacting the gelation bath. With increasing polymer solution concentration (and, therefore, increasing viscosity), the sphere formation occurs slowly, requiring a larger distance between the needle tip and gelation bath [17,23,24].

Concentration alone influenced uniformity most (*p* = 0.0227), as higher concentrations produce a greater number of round microspheres with a narrower size distribution. This can be explained by a higher extent of chain entanglement which leads to an even distribution of droplets during electrospray. The relationship between flow rate and concentration (*p* = 0.0644) greatly impacts uniformity, especially at a low flow rate and high concentration. While the exact details of this phenomenon are still being investigated, flow rates that are too high or too low result in a less stable flow, and therefore increased variability in size [16,22,23]. Based on the analysis, the optimized conditions were determined to be pectin solution concentration of 6% (pH ≈ 4, conductivity = 297.8 µs/cm), voltage of 21 kV, distance of 10 cm, and flow rate of 8 mm hr^−1^.

### 3.2. Influence of Gelatin Concentration on Microsphere Coating

**Figure 2:**
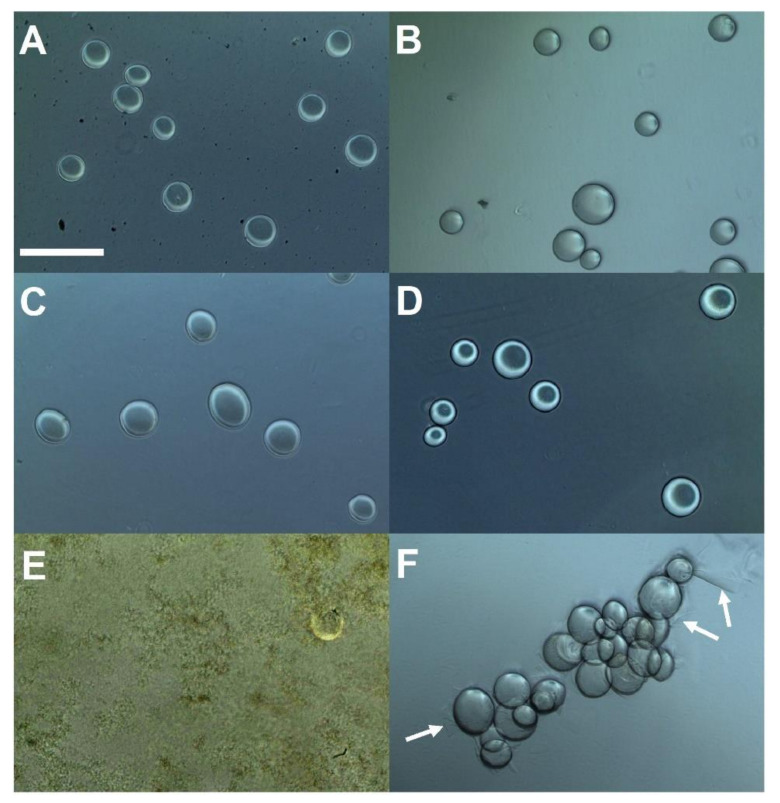
Optical microscope images of microspheres: (**A**) PM in DI water; (**B**) GCM in DI water; (**C**) GCEM in DI water (0.75% gelatin); (**D**) GCEM in PBS (0.75% gelatin); (**E**) PM in PBS; (**F**) microsphere clumping in 1% gelatin solution. Arrows indicate gelatin aggregates. The scale bar represents 500 µm.

Optical microscopy was used to study the morphology of the microspheres after different modifications. Figure 2A–C shows optical microscopy of PM, GCM, and GCEM. Calcium-pectin microspheres are not stable in physiological conditions, such as phosphate-buffered saline (PBS), because they tend to swell and rupture due to the loss of Ca^2+^, as shown in Figure 2E. Because pectin is a polyanion, molecules with a large number of positively charged residues, like gelatin, can be used to form polyelectrolyte complexes that stabilize the microsphere structure. Moreover, gelatin is favored in tissue engineering because of its biodegradability and enhanced cell binding abilities associated with its RGD sequence. The RGD motif is considered a minimal binding domain for recognition by cell membrane integrins, including αvβ3, α5β1, and αIIbβ3. Integrin-RGD binding allows integrins to associate with the actin cytoskeleton and aggregate, forming focal adhesion structures which present structural links between the ECM and cell skeleton to regulate cell adhesion and migration. These adhesive structures also activate distinct signaling pathways that can regulate transcriptional factor activity and direct major cell functions such as migration, proliferation, and differentiation [25]. 

Various gelatin concentrations (0.5–2%) were used to coat the microspheres *via* incubation. Concentrations of 0.5% and 0.75% caused uniform coating of microspheres, with no evidence of microspheres clumping. Concentrations exceeding 1% caused clumping of microspheres (Figure 2F). This phenomenon may be attributed to the fact that localized gelation occurred, as crosslinking may occur among the gelatin molecules residing on the surfaces of adjacent microspheres. Therefore, a gelatin concentration of 0.75% was chosen for further investigation.

### 3.3. Size and Surface Analysis of Microspheres

**Figure 3:**
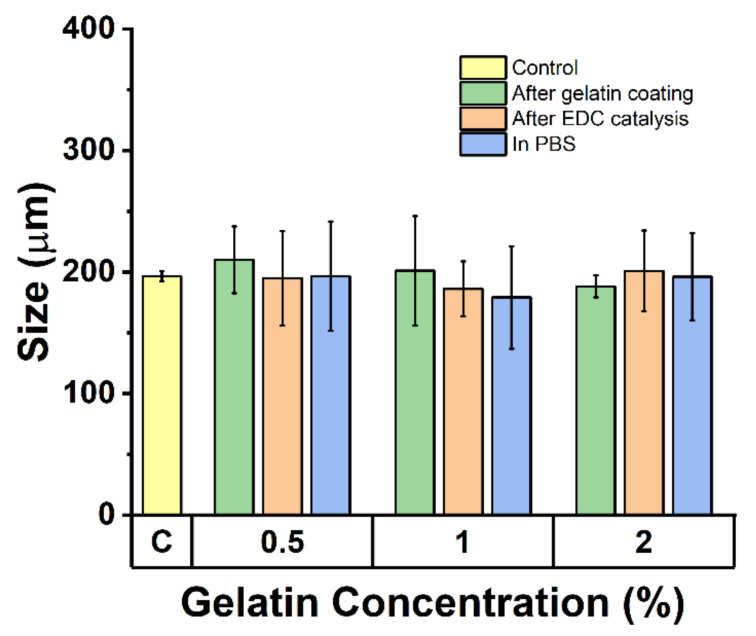
Microsphere size changes at various processing stages.

**Figure 4:**
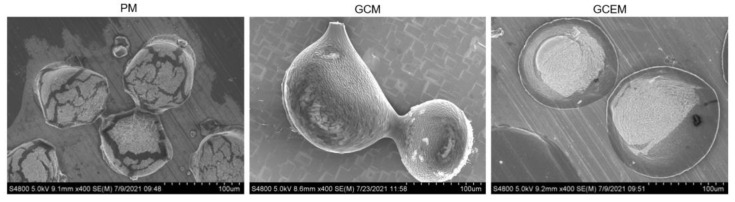
Scanning electron microscope (SEM) images of microspheres.

Images taken using the optical microscope were used for size analysis. At least 40 microspheres were analyzed per sample, employing NIH ImageJ software. As shown in Figure 3, the size of microspheres did not change significantly during gelatin coating or EDC catalysis, regardless of gelatin concentration. The SEM images (Figure 4) show how the microsphere surface morphology changes as the microspheres proceed from having cracks and surface irregularities to having a smoother surface upon gelatin coating and EDC catalysis. 

**Figure 5:**
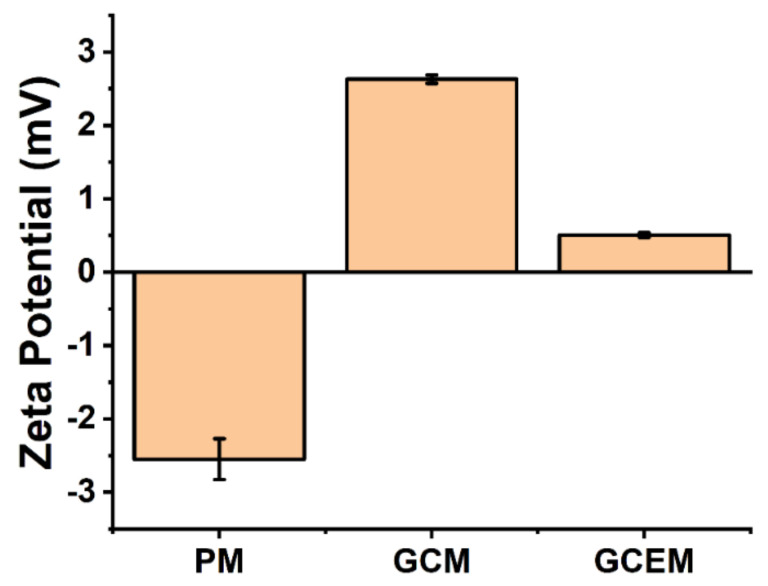
Zeta potential changes of the microspheres throughout the process.

Microspheres with a positively charged surface show potential for cell adhesion and proliferation, as negatively charged cell membranes can attach to positively charged microspheres through electrostatic interactions [26,27]. Gelatin-coated microspheres showed positive surface charges, as expected (Figure 5). EDC catalysis caused a decrease in the positivity of surface charge due to the formation of amide bonds (i.e., losing amino groups, the main contributor to the positive surface charge).

### 3.4. Chemistry of Microspheres

**Scheme 3:**
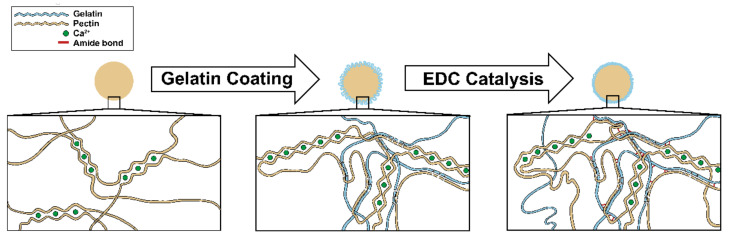
Structure of microspheres at each stage during production process.

**Figure 6:**
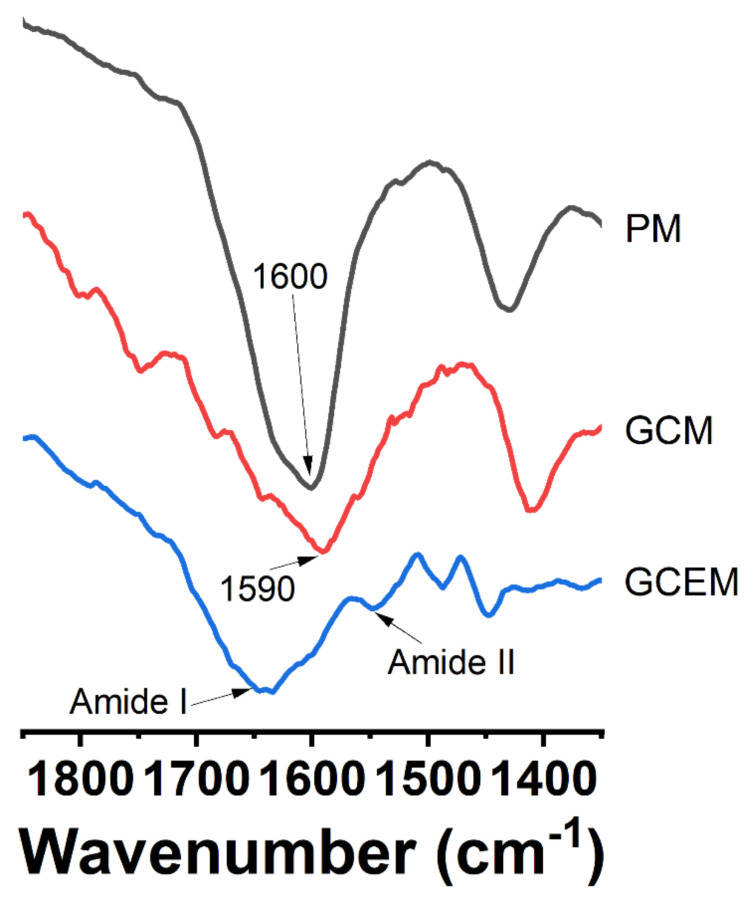
ATR−FTIR spectrum of microspheres at various processing stages.

During gelatin coating, pectin-gelatin complexes were formed at the surface of the microspheres. The carboxyl group of pectin and amino groups of gelatins contribute to the formation of these complexes (as shown in Scheme 3). When it comes to EDC catalysis, amide bonds are formed predominantly between the carboxyl groups of pectin and the amino groups of gelatin. Figure 6 shows the ATR-FTIR spectra of the three samples throughout the various processing stages (the full spectrum is shown in Figure S1). Regarding the calcium-pectin microsphere spectrum, the broad peak around 1600 cm^−1^ is due to COO^−^ groups, while the peak at 1734 cm^−1^ is due to the carbonyl groups of the methylated portions [28]. When it comes to the gelatin-coated microsphere spectrum, characteristic peaks of both pectin and gelatin can be observed. The broad peak around 1590 cm^−1^ is attributed to the COO^−^ of pectin and amide I and II regions of gelatin (1628 cm^−1^ and 1528 cm^−1^, respectively). Upon EDC catalysis, the amide I and II regions became more pronounced, as shown in the spectrum, which can be explained by the formation of amide bonds (changes in N-H bending and C=O stretching).

### 3.5. Bioprintability of Bioink with Microspheres

**Figure 7:**
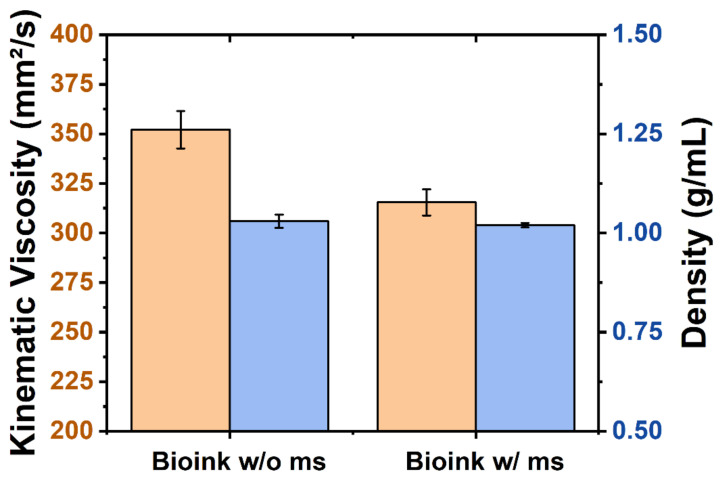
Viscosity and density of bioink with and without microspheres at 4 °C.

The kinematic viscosity and density of bioink with and without gelatin-coated pectin microspheres did not show a significant change (Figure 7). Because of the Pluronic^®^ F-127, the viscosity of the bioink is temperature-dependent. The temperature-dependency can be beneficial when it comes to bioprinting applications. At 4 °C, the kinematic viscosity for bioink without and with microspheres was 352.09 ± 9.41 mm^2^ s^−1^ and 315.45 ± 6.61 mm^2^ s^−^^1^, respectively, a 10.40% decrease upon the incorporation of microspheres. Increasing the temperature to 20 °C, the kinematic viscosity for bioink without and with microspheres was 421.68 ± 4.32 mm^2^ s^−1^ and 376.83 ± 0.76 mm^2^ s^−1^, separately (10.64% decrease). The density for bioink with and without microspheres was 1.030 ± 0.017 g/mL and 1.020 ± 0.006 g/mL, respectively, a 0.99% decrease. 

**Figure 8:**
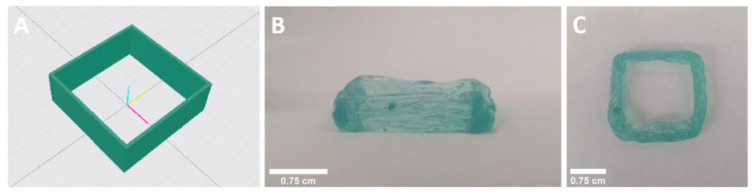
Top (**A**) and side (**B**) view of a bioprinted scaffolds containing GCEM with a screenshot of the generated G-code from the CAD file (**C**).

**Figure 9:**
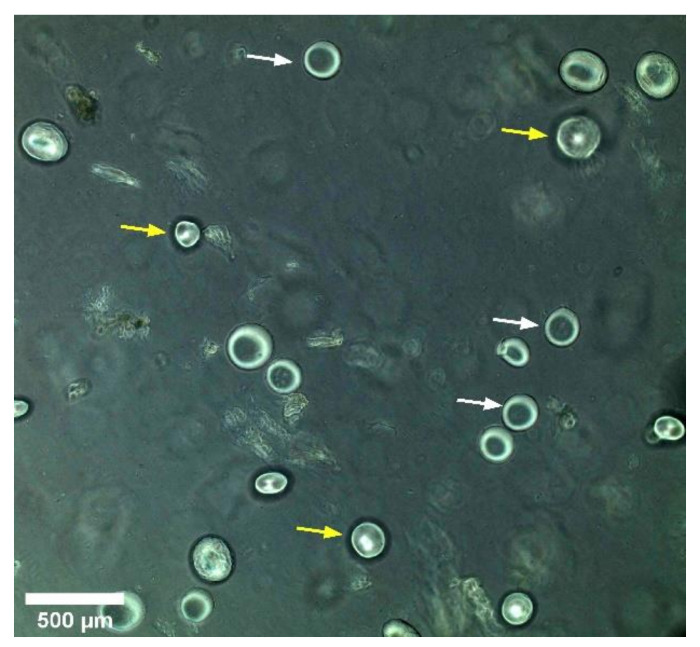
Microsphere distribution within bioprinted scaffold. Both focused (white arrow) and unfocused (yellow arrows) microspheres are depicted.

Upon microsphere incorporation into bioink, the printing occurred smoothly, and no negative effects were observed. Figure 8 shows a square, frame-shaped scaffold that was bio-printed using the bioink with microspheres. Food coloring was utilized to enhance the contrast of the visualization (McCormick^®^ Assorted NEON! Food Colors & Egg Dye; Baltimore, MD, USA). Figure 9 depicts the distribution of microspheres in bioink.

## 4. Conclusions

Gelatin-coated pectin microspheres show promise for tissue engineering applications. When it comes to the production of the calcium-pectin microspheres (i.e., PM) for coating, the optimization process showed that microsphere diameter was predominantly impacted by flow rate, microsphere roundness was most significantly influenced by concentration, and uniformity was primarily affected by concentration. The size of the microspheres remained relatively stable throughout the entire process, and the microspheres exhibited a positive surface charge after gelatin coating and EDC catalysis. The positively charged surface, an indication of successful gelatin coating, is favorable for tissue engineering applications. Moreover, successful gelatin coating and EDC catalysis were confirmed by FTIR and SEM analysis. When incorporated into bioink for scaffolding, the microspheres distributed evenly and did not display any negative effects on bioprintability (e.g., demonstrated through viscosity and density measurements). Future studies could include biocompatibility testing, different methods of crosslinking, such as transglutaminase catalysis, and encapsulation of bioactive compounds into the microspheres to investigate controlled release capabilities. Moreover, stability and degradability of the microspheres will be explored to customize the composition of microspheres for bioink design. 

## Figures and Tables

**Table 1:** Factors and ranges for experimental design.

Factor	Range
Concentration (%)	3.5–6
Voltage (kV)	12–22
Flow rate (mm h^−1^)	5–30
Distance (cm)	5–10

## Data Availability

The data that support the findings of this study are available from the corresponding author upon reasonable request.

## Supplementary Materials

The following are available online at https://www.mdpi.com/article/10.3390/polym13193339/s1, Figure S1: FTIR spectra (400–4000 cm^−1^) of calcium-pectin microspheres (PM), microspheres after gelatin coating (GCM), and GCM after EDC catalysis (GCEM).
