# Supplementary material for: Development of Gelatin-Coated Microspheres for Novel Bioink Design
Source: Polymers (Basel). 2021 Sep 29;13(19):3339. doi: 10.3390/polym13193339 (PMC8512326; doi:10.3390/polym13193339)
Supplement: Supplementary file 1 [file polymers-13-03339-s001.zip › polymers-1376445-supplementary.pdf]

Supplimentary Materials

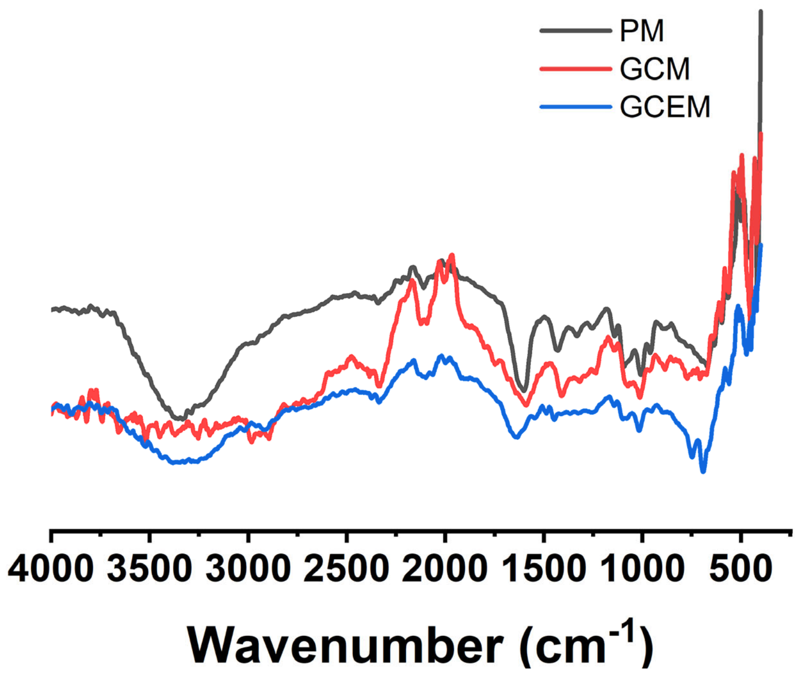

Figure S1: FTIR spectra (400-4000 cm^{-1}) of calcium-pectin microspheres (PM), microspheres after gelatin coating (GCM), and GCM after EDC catalysis (GCEM).
